# Dramatic Enhancement of Rare-Earth Metal–Organic Framework Stability Via Metal Cluster Fluorination

**DOI:** 10.1021/jacsau.2c00259

**Published:** 2022-08-09

**Authors:** Matthew S. Christian, Keith J. Fritzsching, Jacob A. Harvey, Dorina F. Sava Gallis, Tina M. Nenoff, Jessica M. Rimsza

**Affiliations:** † Geochemistry Department, Sandia National Laboratories, Albuquerque, New Mexico 87123, United States; ‡ Organic Materials Science Department, Sandia National Laboratories, Albuquerque, New Mexico 87123, United States; § Nanoscale Sciences Department, Sandia National Laboratories, Albuquerque, New Mexico 87123, United States; ∥ Material, Physical, and Chemical Sciences, Sandia National Laboratories, Albuquerque, New Mexico 87123, United States

**Keywords:** metal−organic frameworks, NMR, density functional theory, fluorine, GIPAW

## Abstract

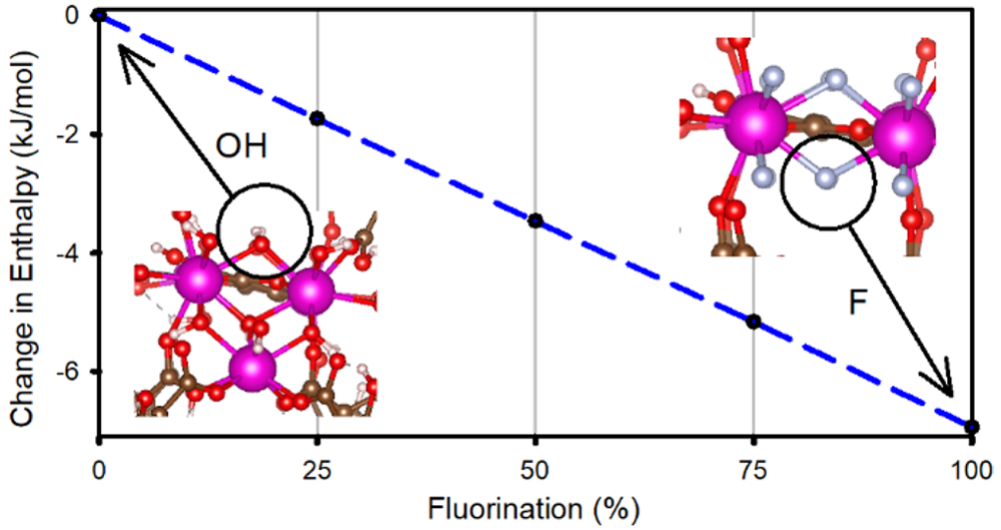

Rare-earth polynuclear metal–organic frameworks (RE-MOFs) have demonstrated high durability for caustic acid gas adsorption and separation based on gas adsorption to the metal clusters. The metal clusters in the RE-MOFs traditionally contain RE metals bound by μ_3_–OH groups connected via organic linkers. Recent studies have suggested that these hydroxyl groups could be replaced by fluorine atoms during synthesis that includes a fluorine-containing modulator. Here, a combined modeling and experimental study was undertaken to elucidate the role of metal cluster fluorination on the thermodynamic stability, structure, and gas adsorption properties of RE-MOFs. Through systematic density-functional theory calculations, fluorinated clusters were found to be thermodynamically more stable than hydroxylated clusters by up to 8–16 kJ/mol per atom for 100% fluorination. The extent of fluorination in the metal clusters was validated through a ^19^F NMR characterization of 2,5-dihydroxyterepthalic acid (Y-DOBDC) MOF synthesized with a fluorine-containing modulator. ^19^F magic-angle spinning NMR identified two primary peaks in the isotropic chemical shift (δ_iso_) spectra located at −64.2 and −69.6 ppm, matching calculated ^19^F NMR δ_iso_ peaks at −63.0 and −70.0 ppm for fluorinated systems. Calculations also indicate that fluorination of the Y-DOBDC MOF had negligible effects on the acid gas (SO_2_, NO_2_, H_2_O) binding energies, which decreased by only ∼4 kJ/mol for the 100% fluorinated structure relative to the hydroxylated structure. Additionally, fluorination did not change the relative gas binding strengths (SO_2_ > H_2_O > NO_2_). Therefore, for the first time the presence of fluorine in the metal clusters was found to significantly stabilize RE-MOFs without changing their acid-gas adsorption properties.

## 1 Introduction

Metal–Organic frameworks (MOFs) have demonstrated acid gas separation and adsorption capabilities based on strong binding between the gas molecule and the metal cluster.^1−13^ These porous MOF structures have large surface areas that can be tailored for specific gas compositions through a judicious selection of organic linkers and metal clusters that preferentially adsorb target gas molecules.^14−18^ An example of the tunability of MOF structures is the incorporation of rare-earth elements (REs) that have high coordination numbers for gas adsorption and tunable magnetic and photoluminescence properties ideal for industrial gas-sensing applications·^19−21^

The properties of RE-based MOFs have been studied extensively both experimentally and computationally. A common RE polynuclear MOF family uses 2,5-dihydroxyterephatalic acid (DOBDC) as the organic linker.^12,21,22^ These RE-DOBDC MOF structures have been found to be comprised of metal clusters with μ_3_–OH groups connected by either mono- or bidentate-bound DOBDC linkers.^23,24^ During synthesis, a 2-fluorobenzoic acid (2-fba) modulator is used to slow nucleation kinetics and control growth of the desired crystalline structure.^2,21,25^ The modulator concentrations in synthesis mixtures can be large relative to the concentrations of the metal salts and the linker. For example, Henkelis et al. successfully synthesized the entire series of Ln-DOBDC MOFs using salt/linker/modulator ratios between 1:1.58:7.98 (Lu-DOBDC) and 1:1.46:21.86 (La-DOBDC).^2^ These RE-DOBDC MOFs have structural and topological similarities to UiO-66.^23^ A recent manuscript by Vizuet et al. reported the possible presence of fluoro bridging groups in metal clusters of Ho-UiO-66 analogues, which they attributed to the use of a fluorinated modulator.^26^

Fluorine incorporation into MOF crystal structures is well-documented,^27−31^ though incorporation has typically been through fluorine modification of the linker to increase specific gas adsorption.^28,29^ The incorporation of fluorine in the metal cluster in MOFs has not been commonly studied. One rare example is a fluorinated cobalt cage crystal structure synthesized and analyzed for H_2_ and CO_2_ uptake^32,33^ as well as for lithium storage in Mn-UiO-66.^28^ However, it is unclear if the incorporation of fluorine into the metal cluster impacts the thermodynamic stability, structural features, and gas adsorption properties of the RE-MOF structures.

Therefore, we identified thermodynamic stabilization of RE-MOFs through fluorine incorporation into RE polynuclear metal clusters and validated our results through the use of computational and experimental NMR spectroscopy. Finally, the potential impact on gas adsorption is calculated, identifying a minimal impact on the application of these materials for acid gas separations.

## 2 Methods

### 2.1 Computational Methods and Formation Enthalpy Calculation

The basis for all hydroxyl and fluorine structures came from experiment; Zr-UiO-66[REF], Eu-DOBDC,^34^ and Eu-TCPB.^15,35^ Each structure underwent four calculations using the Vienna Ab initio Simulations Package (VASP)^36−38^ code to optimize the structure, which allowed the atomic position to change while the volume was kept constant, to then optimize the atomic positions and the cell volume simultaneously, and to then, finally, optimize the atomic positions only. All calculations used large core potentials^39^ for the RE(III) oxidation state and a 500 eV cutoff using the PBEsol exchange-correlation functional.^40^ van der Waals interactions were included using the Grimme D3 dispersion correction.^41,42^ Each calculation had an energy convergence criterion of 10^–6^ eV with a γ-point *k-*point mesh using the real-space algorithm. A final single-point calculation using the *k*-space approach was used to calculate formation enthalpies. Formation enthalpies *H* were calculated using OQMD fitted potentials^43^ such that
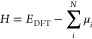
1where *E*_DFT_ is the MOF density functional theory (DFT) calculated energy, and μ_*i*_ is the fitted atomic potential for element *i*. To condense the change in crystal lattice angles from three variables (α, β, and γ), the absolute lattice angle change from fluorine addition was calculated with the following equation.
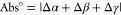
2

### 2.2 Computational NMR with the GIPAW Method

The Y-DOBDC MOF structures that were minimized using VASP were used as the input structure for the NMR calculations. A single-point self-consistent field (SCF) calculation was performed using Quantum Espresso,^44^ an open source electronic structure code. Norm-conserving pseudopotentials^45^ with the generalized gradient approximation in the form of Perdew, Burke, and Ernzerhof (PBE)^46^ were implemented. An energy cutoff of 90 Ry and a 1 × 1 × 1 k-point matrix were used with a total energy convergence threshold of 10^–10^, a force convergence threshold of 10^–8^, and a self-consistent energy threshold of 10^–8^. Computational NMR chemical shifts were calculated using the gauge-including projector augmented wave (GIPAW) method.^47^ The GIPAW method is used for the calculation of electric field gradient and absolute shielding tensors (δ_iso_). Refer to ref (48) for a more complete description of the GIPAW method. Absolute shielding tensors for the computational structures were calculated from fully converged all-electron calculations. The experimental (δ^exp^) and calculated (δ^calc^) ^19^F and ^13^C NMR chemical shifts are related to the isotropic component of the chemical shielding tensor, σ_iso_, using
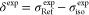
3.a
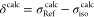
3.bwhere σ_ref_ is the isotropic component of the reference chemical shielding tensor. Variation in the experimental chemical shift (or chemical shielding) is proportional to the variation in the chemical shift obtained from DFT calculations.^49^
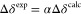
4.a
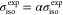
4.b

The reference standard for ^1^H NMR experiments is tetramethylsilane (TMS), but σ_ref_^exp^ or σ_ref_^calc^ are not equivalent. For referencing of calculated ^19^F σ values, the proportionality between α and σ_ref_^calc^ was determined through a linear relationship between experimentally reported chemical shifts (δ_F_^exp^) to calculated chemical shieldings (σ_F,iso_^calc^) via the following equation.
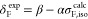
5

Several previous computational studies have been published on the GIPAW method of calculating NMR spectra for crystalline fluorides, which have included values for α and β, see Table S1.^50−53^ On the basis of the variety of crystalline fluorides used for indexing, α = −0.80 and β = 89 ppm were used to reference calculated ^19^F δ_iso_ values, consistent with the work of Sadoc et al.^52^ The same referencing scheme has been previously used by the authors for referencing of ^19^F δ_iso_ values for fluorinated graphene systems.^54^

### 2.3 Experimental NMR Methods

Solid-state NMR experiments were performed on a Bruker 600 MHz Avance II or a 400 MHz Avance III HD spectrometer. The direct excitation Hahn echo experiments were conducted on a 600 MHz instrument using a 1.3 mm HX magic-angle spinning (MAS) probe, with the ^1^H channel tuned down to ^19^F. The MAS frequency was regulated at 62.5 kHz. Typical ^1^H, ^19^F 90° pulse lengths were 1.6 and 1.9 μs, respectively. The ^19^F experiments were collected after a 10-rotor period Hahn echo to minimize probe head background. The experiments conducted at 400 MHz for ^1^H used a 2.5 mm H/FX MAS probe with low ^19^F probe head background. The MAS frequency was regulated at 33.333 kHz. Typical ^19^F 90° pulse lengths were 1.75 μs. A recycle delay of 3 s was used for ^19^F echo experiments and 1 s for two-dimensional (2D) exchange and CODEX experiments. ^19^F shifts were externally referenced by setting the peak from Teflon to −122 ppm on the CFCl_3_ scale.

The 2D ^19^F–^19^F exchange experiments were run at a 33 kHz MAS rate and static magnetic field of 9.4 T. The indirect dimension was run to 0.72 ms with 48 complex points. The area of the cross peak was estimated by fitting a two-peak model to the slice through the indirect dimension (−69.8 ppm). The buildup curve was fit to a stretched exponential model. The stretched exponential was interpreted as an approximation to multiexponential overlap, that is, the overlap of several exponential build-up rates, between spins separated by (slightly) different distances. The direct polarization CODEX experiments were also run at a 33 kHz MAS rate and static magnetic field of 9.4 T. The mixing time *t*_m_ was varied from 30 μs to 12 ms, but the exchange period was fixed at 8*t*_r_. Again the data was fit to a stretched exponential model, interpreted as an approximation to multiexponential overlap. The time constant of the exponential decay is ∼4 times faster than the buildup in the exchange experiment; this is due to the recoupling pulses in the CODEX experiment that partially recouple the dipolar couplings during MAS.

## 3 Results and Discussion

**Figure 1:**
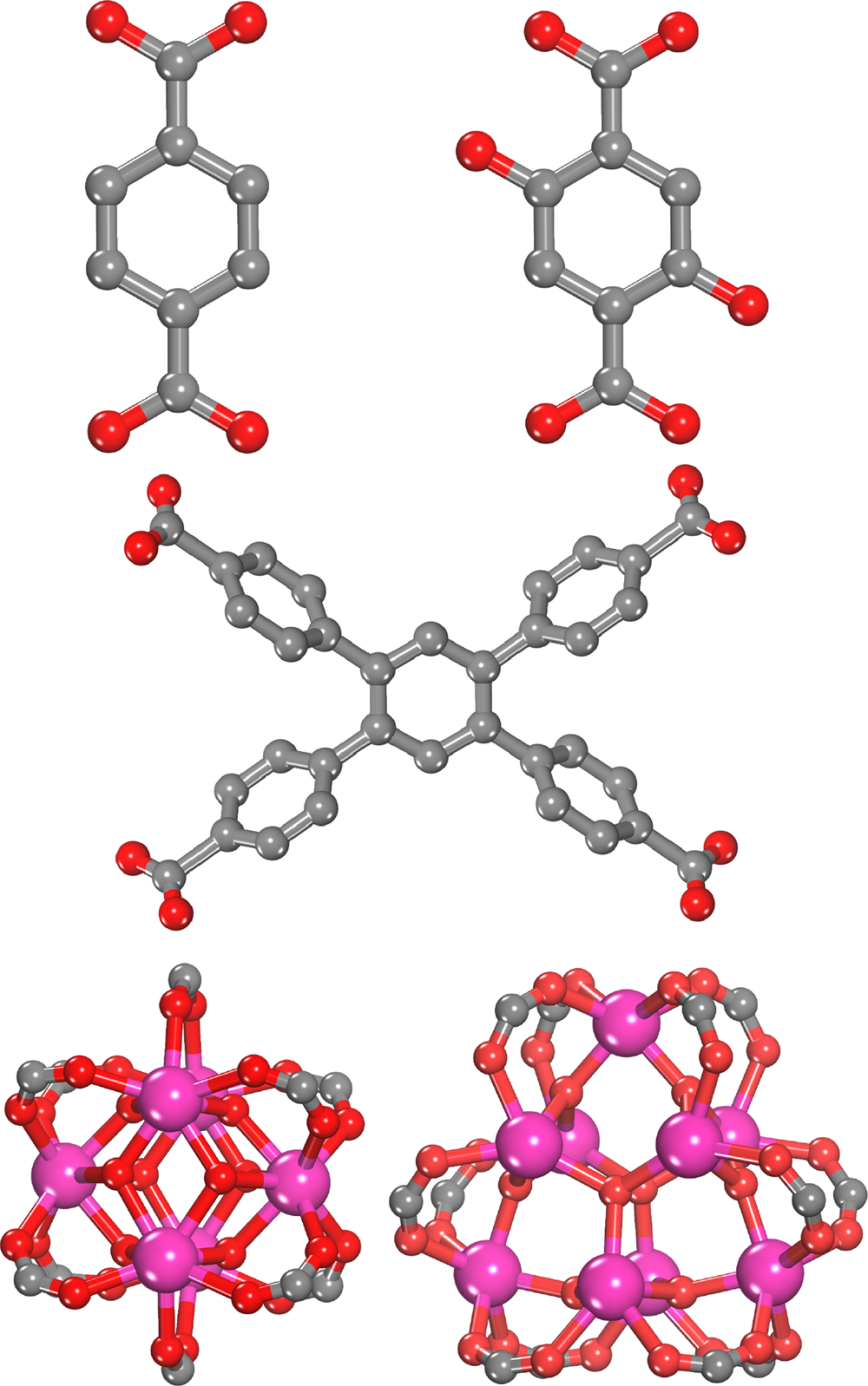
Linkers and metal clusters for formation of RE—MOF structures. Linkers: (upper left) 1,4-benzenedicarboxylate (BDC) linker in UiO-66, (upper right) 2,5-dihydroxyterephthalic acid (DOBDC) linker in RE-DOBDC, (middle) 1,2,4,5-tetra(4-carboxyphenylbenzene (TCPB) linker in RE-TCPB. Polynuclear metal clusters in (bottom left) RE-DOBDC and (bottom right) RE-TCPB. Atom colors: C = gray, O = red, and RE = pink. The hydrogen atoms and coordinating solvents were removed for clarity.

Here, for the first time, we identify the thermodynamic impact of fluorine incorporation into RE polynuclear metal clusters. We performed a systematic computational investigation of OH substitution by fluorine in three RE-MOFs with different metal clusters and organic linker compositions: RE-UiO-66, RE-DOBDC, and RE-1,2,4,5-tetra(4-carboxyphenylbenzene) (RE-TCPB) structures.^15,35^ The three linkers and the metal clusters are included in Figure 1.

The three RE-DOBDC-based MOFs were generated from experimental crystal structures^23,35,55^ with five different RE metals (RE = Y, Eu, Tb, Ho, and Yb) as exemplars. Each of these MOF structures varies in the composition of the metal clusters and the number of RE–OH bridges.

**Figure 2:**
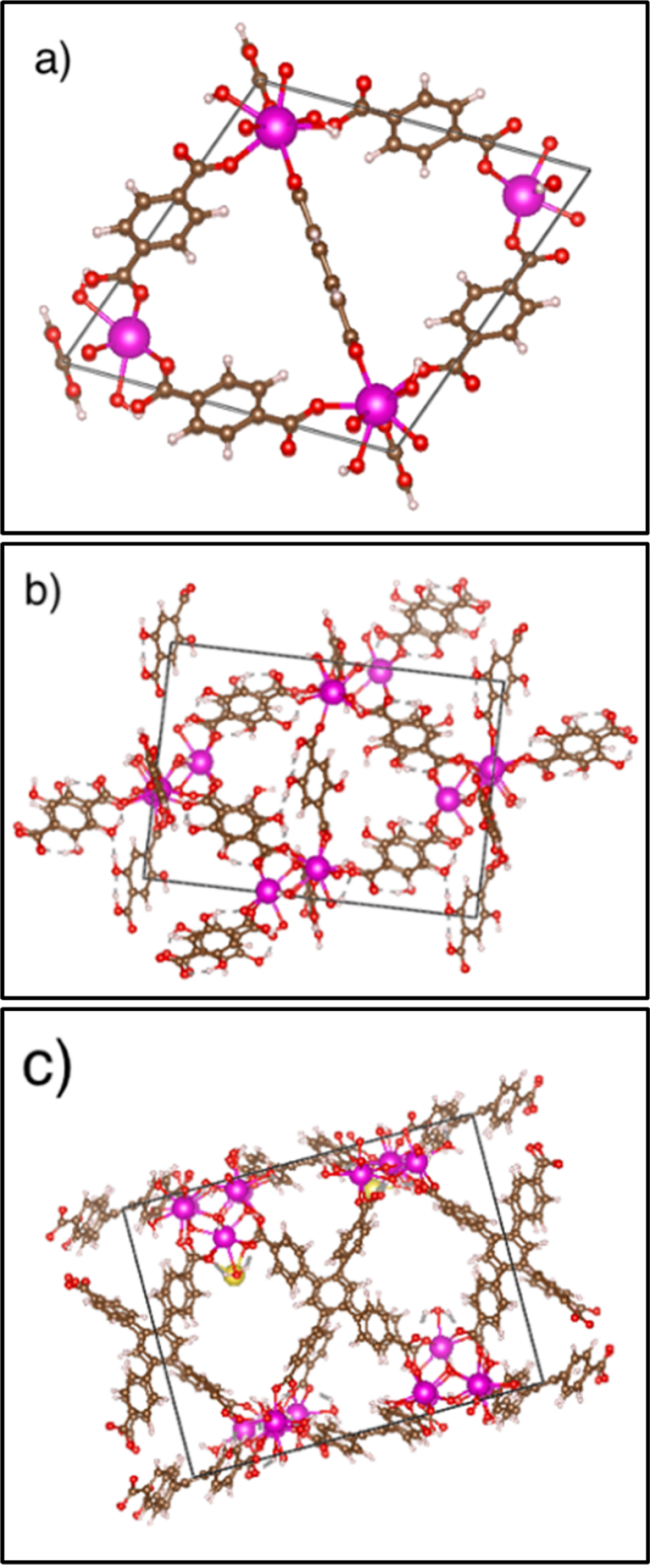
Crystal structures of (a) RE-UiO-66 that has single metal clusters with four hydroxide groups, (b) RE-DOBDC that has two metal clusters with eight hydroxide sites each, and (c) RE-TCPB structures that have two metal clusters with 12 hydroxide sites each. Atom colors: C = gray, H = white, O = red, Na = yellow, and RE = pink.

The RE-UiO-66 structure is comprised of RE hexanuclear clusters bridged by 1,4-benzenedicarboxylate (BDC) linkers. We use a primitive cell that has been previously used to model UiO-66 properties.^56^ It has the smallest volume of the three MOF frameworks studied here, as seen in Figure 2a. The four hydroxyl sites were systematically replaced by fluorine, from 100% hydroxide to 0% hydroxide, with the balance being fluorine atoms, and formation enthalpies were calculated using DFT.

**Figure 3:**
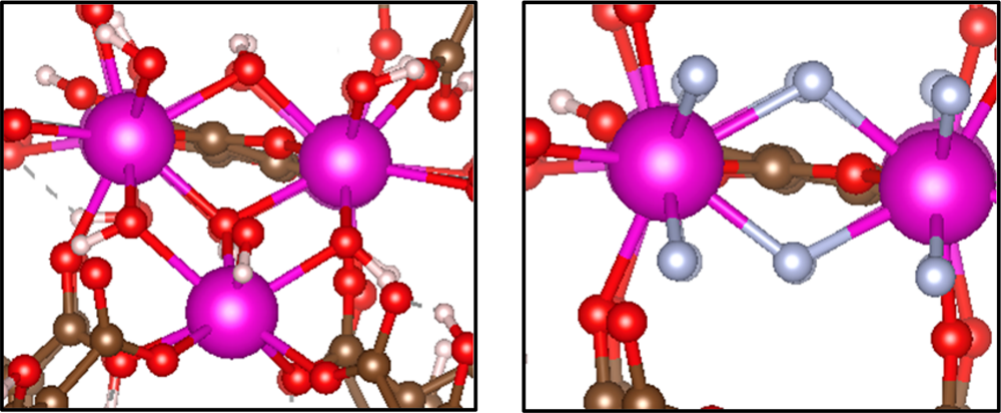
Snapshots of fully hydroxylated RE-DOBDC MOF cluster (left) μ_3_–OH and the fully fluorinated version (right) μ_3_-F. Atom colors: C = brown, F = periwinkle, H = white, O = red, and RE = pink.

The RE-DOBDC structure is comprised of RE hexanuclear clusters bridged by 2,5-dihydroxyterephthalic acid (DOBDC) linkers and contains two metal clusters per unit cell, with eight hydroxyl sites. See a snapshot of the structure in Figure 2b. Here, uniform versus random distributed fluorine substitution in the RE-DOBDC structure was studied for effects on structure stability. Uniform substituted structures were generated by replacing hydroxyls with fluorine one-by-one on a single metal cluster while distributed fluorinated structures were generated by randomly placing hydroxyl groups with fluorine across both metal clusters in the system in 12.5% increments. The result yielded eight different structures with varying fluorination values. See a snapshot of a fully fluorinated metal cluster in the Eu-UiO-66 structure in Figure 3.

The RE-TCPB MOF structure is comprised of RE nonanuclear clusters bridged by 1,2,4,5-tetra(4-carboxyphenylbenzene (TCPB) linkers and contains two metal clusters per unit cell, with 12 hydroxide sites in each metal cluster, as seen in Figure 2c. The large size of the RE-TCPB MOF unit cell limits computational analysis due to time constraints, so only the Eu-TCPB MOFs were simulated. Partial fluorine replacement was achieved by replacing one and two hydroxide(s) on each metal cluster to investigate low doping stability, then replacing two distributed hydroxides with fluorine on each metal cluster, down to 0% hydroxide.

Fluorinated MOFs were then structurally optimized, allowing the cell volume and the atomic positions to change. The periodic DFT calculations used a relaxation protocol, cutoff energies, pseudopotentials, and k-points consistent with previously published simulations of RE-DOBDC MOFs.^21,22,57^ Calculation details are provided in the Supporting Information.

**Figure 4:**
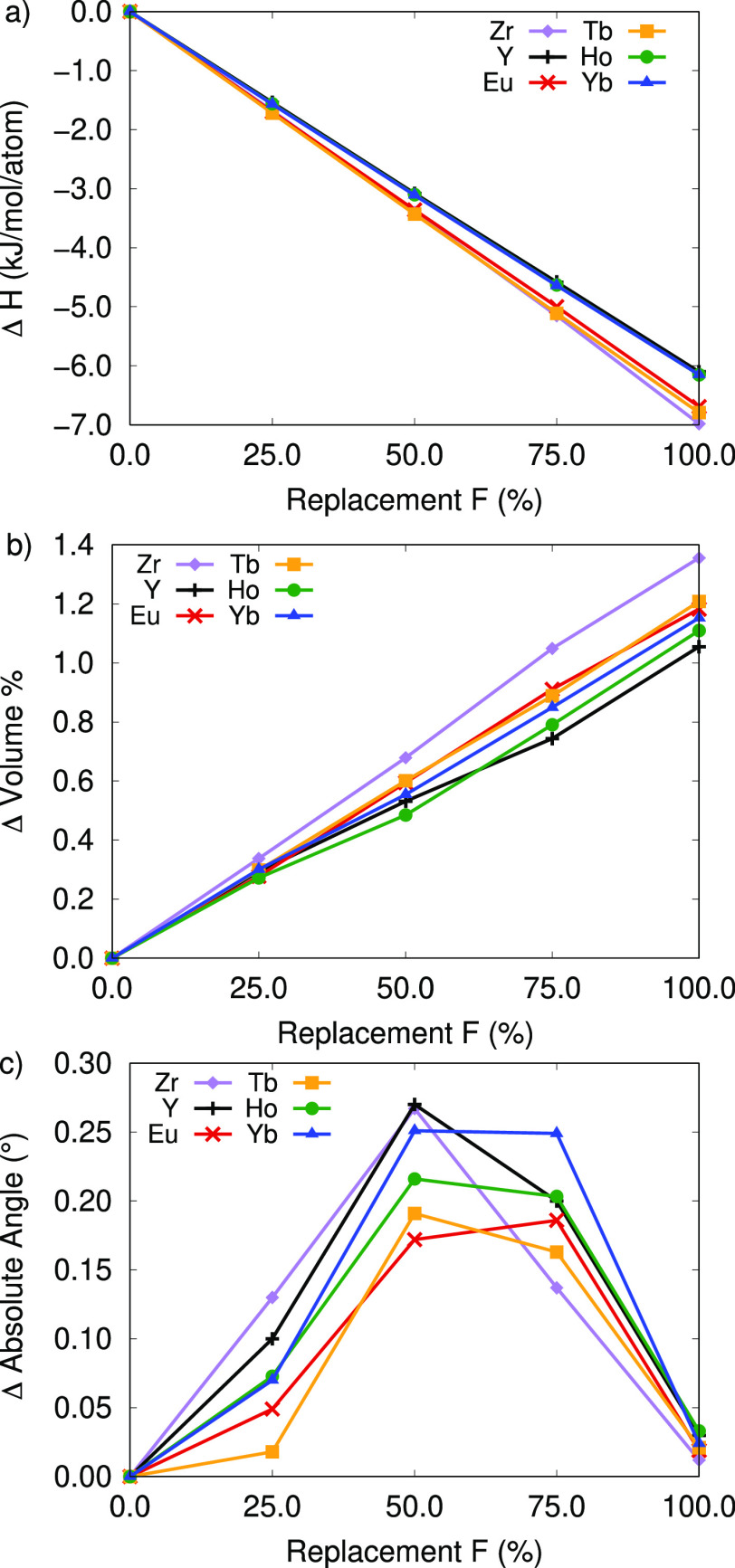
(a) Change in formation enthalpy with inset at 50% F, (b) percent change in volume, and (c) absolute sum in lattice angle change for RE-UiO-66 structures reference to the fully hydroxylated RE-UiO-66 structure.

Results identified that the replacement of hydroxides by fluorine increases the formation enthalpy of the RE-MOF structures and increases the MOF stability. See Figure 4. Note that more negative values indicate greater thermodynamic stability of the system.

For RE-UiO-66, the thermodynamic stabilization is linear with increasing fluorine replacement. As fluorine ions are added to the clusters, the RE-RE bond distances decrease by 0.02 Å due to the introduction of shorter RE-F bond lengths (Re–F = 2.25–2.44 Å vs RE-O = 2.26–2.40 Å). The change in enthalpies is consistent for each metal composition and results in a stabilization of 6–7 kJ/mol per atom relative to the hydroxyl structural analogue. In Figure 4 Y-, Ho-, and Yb-UiO-66 MOF structures exhibited overlapping changes in enthalpy with fluorination, while Eu-, Tb-, and Zr-UiO-66 MOFs were more stabilized by the presence of fluorine in the metal cluster. Zr-UiO-66 has the greatest change in both MOF structure and thermodynamic stability because it has an ∼30% smaller atomic radius compared to the RE metals. This allows for shorter M-F bond lengths and greater van der Waals interactions compared to those in RE-UiO-66.

The change in enthalpies is accompanied by changes in the cell volume and angles of the crystal structures. See Figure 4 and Table S1. The cell volume consistently decreases with increasing fluorination. Additionally, fluorination introduces low levels of distortion in the unit cell by perturbing the α, β, and γ lattice parameters away from 90°, as in the tetragonal unit cell for the 100% hydroxylated crystal structure. The change in the α, β, and γ lattice parameters peaks at a 1:1 hydroxide/fluorine ratio (except for Eu-UiO-66, which has maximum distortion at 75% replacement), indicating the maximum lattice distortion. Despite the distortion in the angles of the unit cell, the thermodynamic stabilization consistently increases with increasing fluorination. Therefore, the thermodynamic stabilization of the MOF from fluorination is due to the shrinking of the unit cell, see Figure 4b, which is supported by the negative change in enthalpy in Figure 4a.

**Figure 5:**
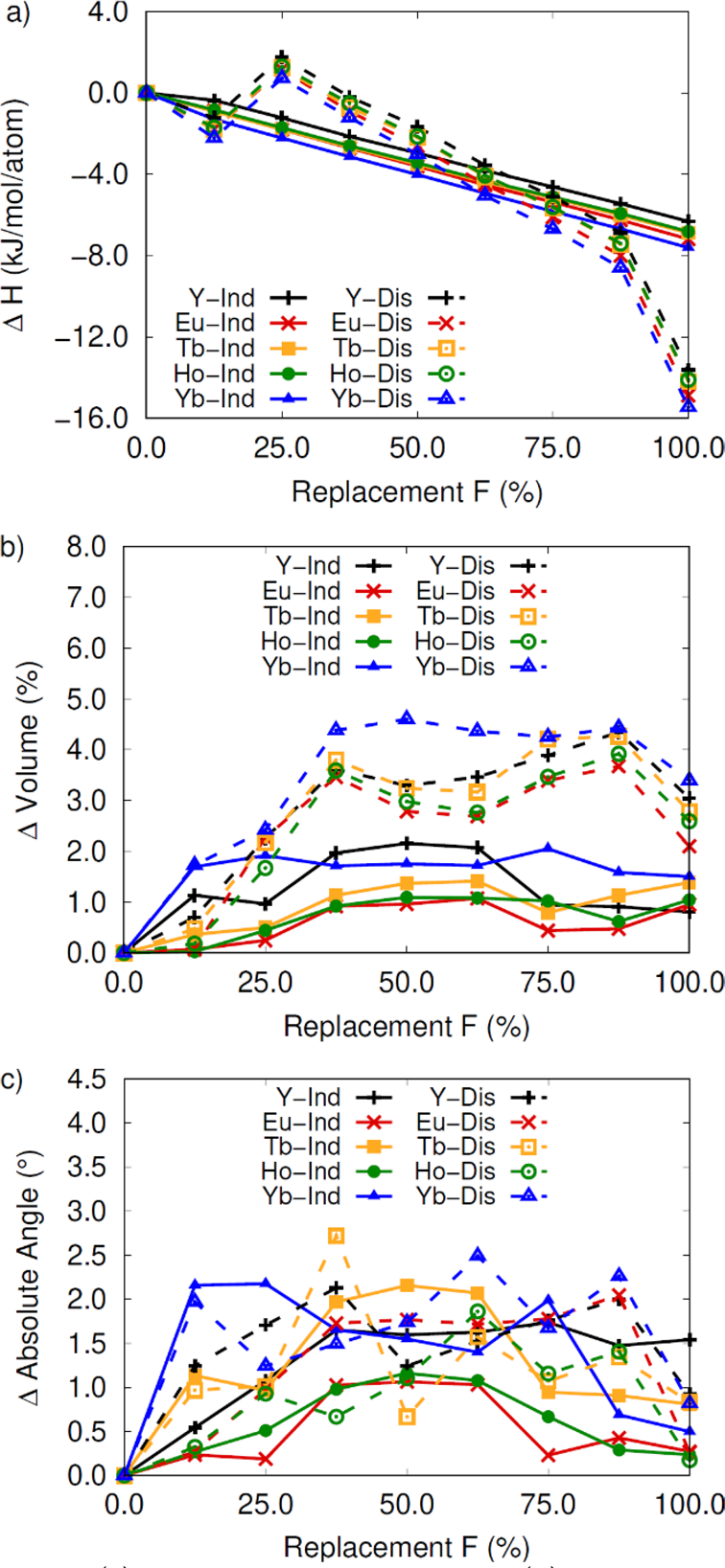
(a) Change in formation enthalpy, (b) percent change in volume, and (c) absolute sum in lattice angle change for RE-DOBDC structures referenced to the fully hydroxylated systems. Solid lines indicate hydroxide replacement on a single cluster in the crystal cell, while a dashed line represents replacement on two metal clusters in the crystal structure.

In the RE-DOBDC MOF, stabilization by fluorination varies based on the distribution of the fluorine atom in the metal clusters. Adding fluorine to only one cluster results in a linear trend as seen in UiO-66, stabilizing the MOF structure, and decreasing the system energy by 6–8 kJ/mol per atom for 100% fluorination (Figure 5, solid lines). However, when fluorine replacement is split between the two metal clusters, changes in the thermodynamic stabilities are not linear, indicating that the fluorine replacement is site-dependent. Thermodynamic stability decreased, with system energies 1–2 kJ/mol per atom higher when 20% of the hydroxyl groups are replaced with fluorine split between the two metal clusters. Yet, when fluorination increases to a 50% split between two metal clusters, the system becomes thermodynamically stable again. From there, fluorine replacement is increasingly favorable on both metal cluster sites until the final thermodynamic stabilization of the structure at 100% fluorination by decreasing the system energy by 15–16 kJ/mol per atom.

The change in thermodynamic stability arises from perturbation of the crystal structure. This is seen in the absolute change in the cell volume lattice angles in Figure 5a,b. When fluorine is substituted on one cluster, the change in cell volume is only 0.5%–2.0%. By comparison, when fluorine is substituted in both metal clusters, the change in volume is much larger for systems with 40%+ fluorine content, between 2.0 and 4.5%. Despite these differences in volume, the amount of distortion in the unit cell is relatively constant, identified by angle changes, see Figure 5c. Therefore, the level of distortion in the unit cell is not impacted by the location of the fluorine, only the cell volume.

Within the metal cluster, the RE-RE interatomic distance decreases by 0.07 Å following fluorination. van der Waals forces vary by *R*^–6^ (*R* = distance between atoms), so that the contraction of the RE-RE distances increases van der Waals interactions. A similar effect is seen for the RE-F distances that decrease by 0.1 Å between the 25% fluorinated and the 100% fluorinated structures. The increase in van der Waals interactions with fluorination increases the thermodynamic stability of the fluorinated systems resulting in clear stabilization of the RE-DOBDC MOF framework with increasing fluorine concentration.

**Figure 6:**
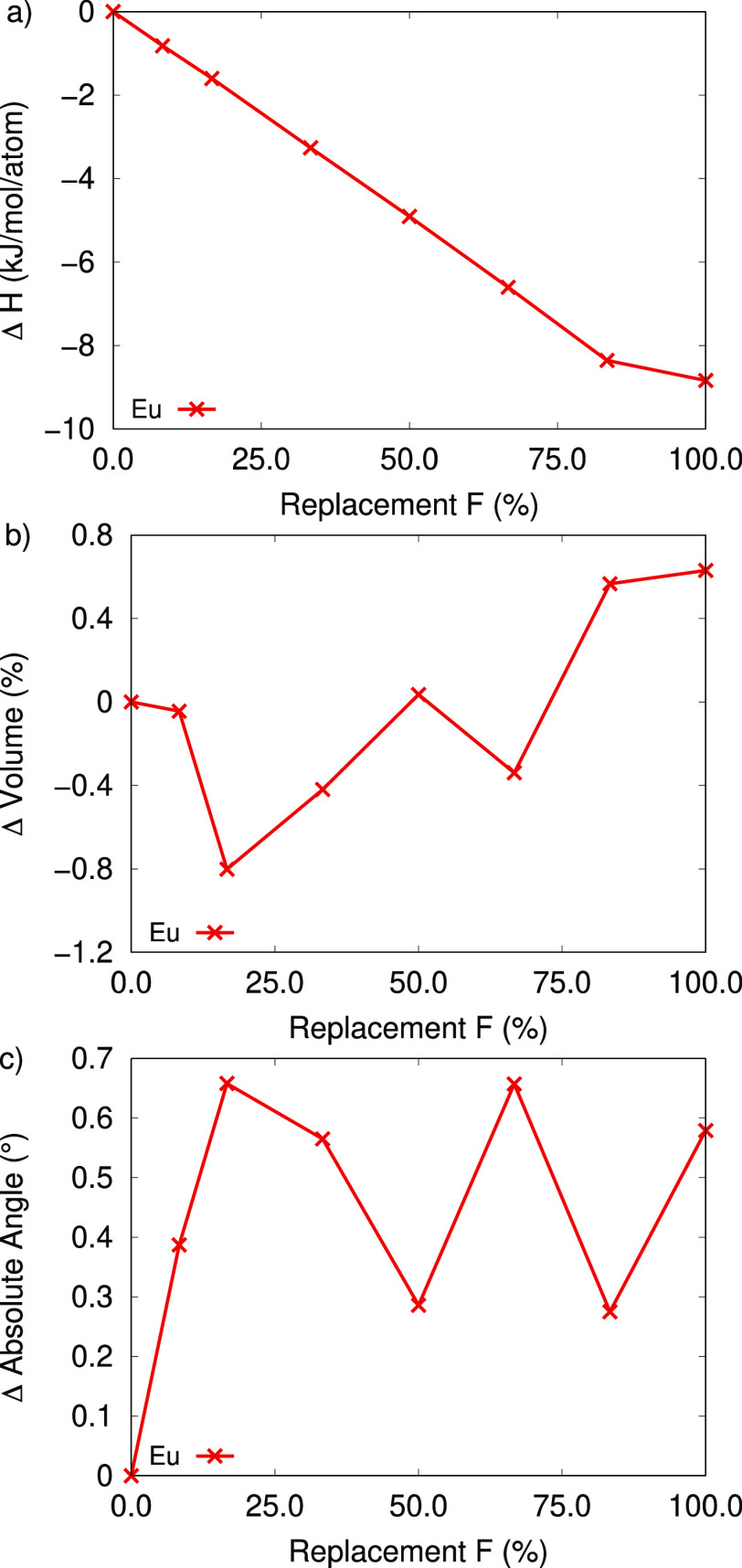
(a) Change in formation enthalpy, (b) percent change in volume, and (c) absolute sum in lattice angle change for Eu-TCPB structures referenced to the fully hydroxylated systems.

Remarkably, the RE-TCPB MOF, the largest MOF unit cell studied here, is also stabilized by inclusion of fluorine atoms in the metal cluster. Fluorination decreases the system energy by 9–10 kJ/mol per atom, as seen in Figure 6a. Interestingly, this is less than RE-DOBDC, and the stabilization effect is linear with respect to fluorine replacement, like UiO-66, despite having structural complexity closer to the RE-DOBDC MOFs. Overall, fluorination of the metal cluster increases the thermodynamic stability of the RE-TCPB MOF, as seen in Figure 6a. The stabilization of the RE-TCPB MOF slows at 83% fluorine replacement along with less change in the volume, see Figure 6b. The change in stabilization rate at the higher fluorination content is due to the size of the metal cluster in the RE-TCPB MOF and the expansion of the metal cluster during fluorination. In the RE-TCPB MOF metal clusters the RE-RE distances increase by 0.08 Å, and RE-F bond lengths increase by 0.12 Å. This is the only RE-MOF in which the RE-RE distances increase during fluorination, which introduces more distortion in the unit cell via perturbing the lattice angles relative to 90°. The absolute change in lattice angle (0.6°) combined with the increase in volume (0.7%) at 100% fluorination decrease the amount of thermodynamic stabilization at these high fluorination rates, see Figure 6c. The consistent stabilization of the RE-MOF structure by fluorine incorporation indicates that this is a persistent effect among RE-MOFs.

The predicted thermodynamic stability of the fluorinated RE-DOBDC MOF structures was validated via computational and experimental NMR spectroscopy. Computational ^19^F NMR spectra were calculated for the 100%, 75%, 50%, and 25% fluorinated Y-DOBDC MOFs using the GIPAW^49^ method in the Quantum-Espresso code.^44^ Structures for NMR calculations used the final relaxed structures from the prior DFT calculations, consistent with data in Figure 4. Chemical shifts were referenced to crystalline fluoride structures used successfully for an analysis of fluorinated-graphene structures.^54^ Additional details are provided in the Supporting Information.

Model results indicated that the ^19^F isotropic chemical shifts (δ_iso_) vary from −56 to −87 ppm, depending on the local chemical environment. Three peaks were seen at −63.0, −70.0, and −87.0 ppm, see a histogram of all ^19^F δ_iso_ in Figure S1, across the four different fluorination levels. The population of these shifts varies as a function of fluorination. The middle ^19^F δ_iso_ peak, at −70 ppm, loses intensity as the fluorination decreases, see Table 1. As a result, the ^19^F δ_iso_ peak at −63 ppm continues to increase in intensity up to 25% fluorination until only two chemical environments are present, one at −63 ppm and one at −87 ppm. Structurally, the ^19^F δ_iso_ varies with the Y–F distances. See Figure S2. This demonstrates that, as the Y–F bond distance decreases, the ^19^F δ_iso_ becomes less negative. The peak at −87 ppm is from the longest Y–F bond distances of 2.29–2.32 Å. Additionally, the presence of this shift at lower fluorination rate indicates that it is from an isolated fluorine in the metal clusters. The ratio of the peak heights from the calculated NMR data can be used to estimate the amount of fluorination in the metal clusters from experimental ^19^F NMR data.

**Table 1:** Percentage of Calculated ^19^F δ_iso_ Shifts under the Three Identified Peaks (−63, −70, −87 ppm) in the Y-DOBDC MOF Structure

peak No.	^19^F δ_iso_ (ppm)	100% F	75% F	50% F	25% F
**1**	–63	25.0	50.0	62.5	50.0
**2**	–70	75.0	33.3	25.0	0.0
**3**	–87	0	16.6	12.5	50.0
ratio peak 2/peak 1		3.0	0.67	0.4	0.0

**Figure 7:**
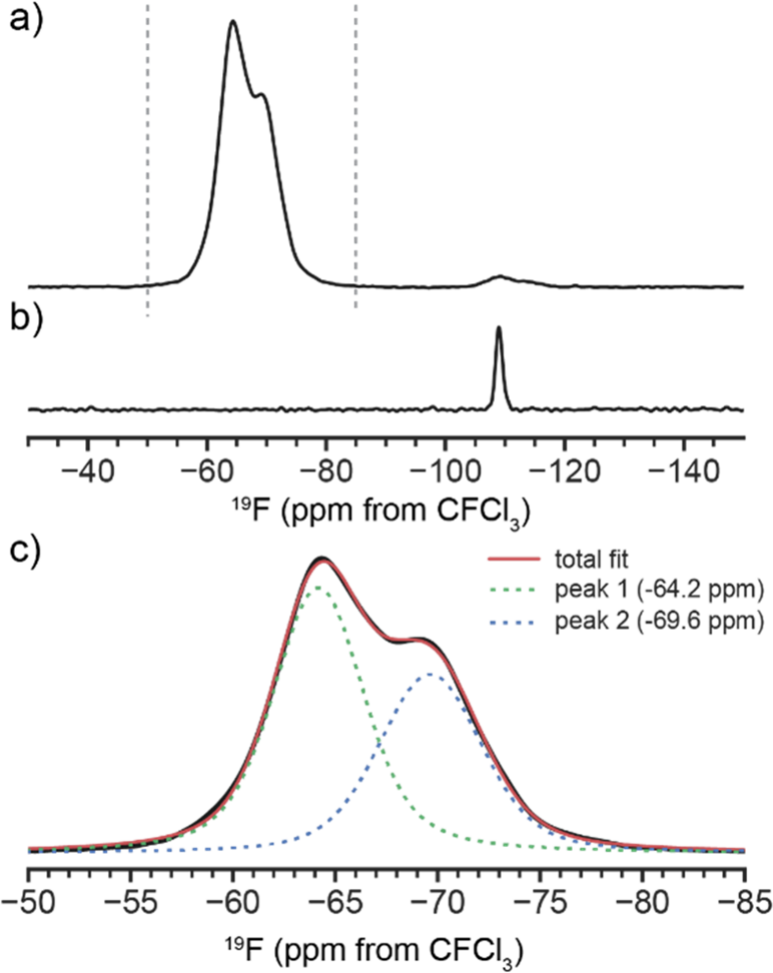
^19^F (60 kHz MAS, 14.1 T B_0_) NMR spectra of (a) Y-DOBC-MOF and (b) crystalline 2-fba. (c) Deconvolution of the main peak in (a) with two Voigt peaks. The ratio of peak 2 at −69.6 ppm to peak 1 at −64.2 ppm is 0.75.

Experimental magic-angle spinning NMR was performed on a Y-DOBDC MOF sample that was previously synthesized with the fluorinated modulator.^2^ The experimental ^19^F spectrum in Figure 7 shows two main peaks at −64.2 and −69.6 ppm. There is also a minor peak (3% of the total ^19^F signal) at 109 ppm, assigned to residual 2-fba. The relative ratio of the integral of the −64.2 ppm peak to the second 69.6 ppm peak is ∼0.75. Comparison with the calculated ^19^F δ_iso_ data in Table 1 indicates that the same ratio of peak 1 to peak 2 data would be expected to occur at a fluorination level of ∼75%.

**Figure 8:**
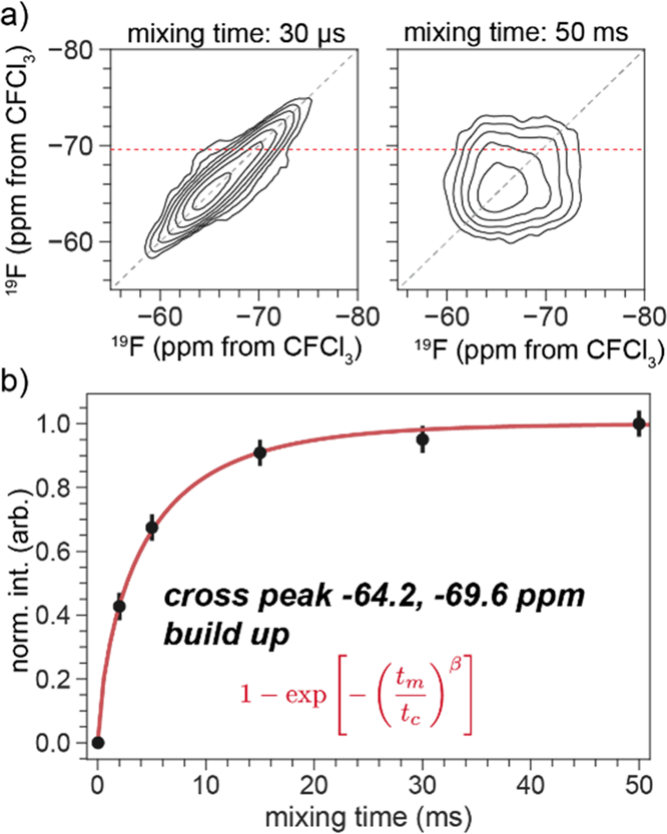
^19^F (33 kHz MAS, 9.4 T B_0_) 2D exchange NMR (a) spectra of Y-DOBC-MOF at different mixing times and (b) buildup of cross peak intensity and fit to a simple exponential model, see Figure S3. The intensities in (b) were determined by deconvolution the cross peak and diagonal peak at the slice shown with the red dashed line in (a). The error bars are at 3σ. Fit parameters are *t*_c_ = 4.4 ± 0.02 ms and β = 0.72 ± 0.05. The 2D contours are plotted starting at 10 times the standard deviation of the noise, in steps of 1.5*x*.

To corroborate the assignment of multiple fluorine sites in the metal cluster, several additional solid-sate NMR explements were performed. 2D Z-exchange NMR spectra show correlation between the two main peaks, seen as off-diagonal cross peak intensity, starting at a 2 ms mixing time (Figure 8). This cross-peak intensity and fast buildup time due to strong dipolar couplings confirms that the two fluorine sites are within ∼5 Å of each other and, therefore, reside in the same metal cluster.

The δ_iso_ of the distinguishable sites nearly overlap. While it is possible to determine the ratio of the peaks from the simple one-dimensional (1D) experiment, without additional data the absolute populations of each site are unknown. Therefore, the Center band-Only Detection of Exchange (CODEX)^58^ NMR experiment was used to determine the number of spins in sites with different molecular orientations and within a short distance to each other.^59^ At long mixing times (*t*_m_) in CODEX experiments, the peaks decay to 1/*n* of the intensity of the *t*_m_ = 0 experiment, where *n* is the number of dipolar coupled spins.

**Figure 9:**
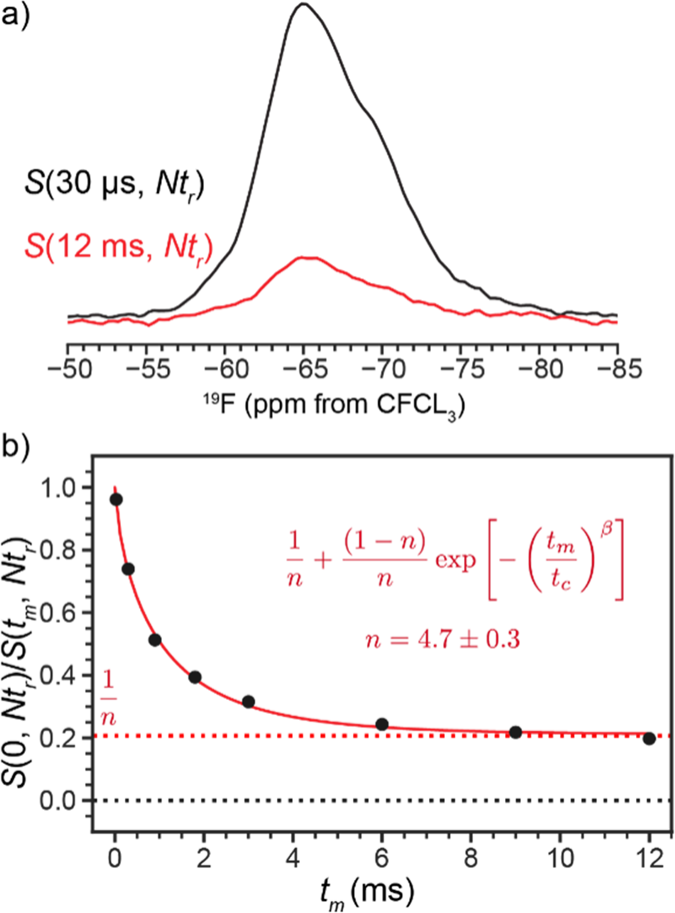
^19^F (33 kHz MAS, 9.4 T B_0_) CODEX NMR (a) spectra at a mixing time *t*_m_ = 30 μs (black), and 12 ms (red). (b) Normalized integral of peak area as a function of *t*_m_ decays to ∼0.2. Fit parameter*s* are *t*_c_ = 1.03 ± 0.06 ms, *n* = 4.7 ± 0.3, and β = 0.72 ± 0.04. The β parameter was introduced because of the of inhomogeneous line shape. The error bars in (b) are plotted at 6 times the standard deviation of the spectrum noise but are obscured by the data markers. The resolution in these spectra collected on a 400 MHz spectrometer is lower than the resolution of the spectra in Figure 7 collected on a 600 MHz instrument.

The CODEX experiment demonstrates that, on average, there are greater than four fluorine atoms per cluster. Figure 9 shows that the signal decays to ∼0.2 in 12 ms, corresponding to 4.7 ± 0.3 (±1σ) nearby (∼5 Å) fluorine spins. Since the Y-DOBDC MOF structures have eight possible fluorine sites, these data indicate a fluorination rate of more than 50%, validating the high stability of fluorine in the metal clusters in the calculation results.

Given the predicted and experimentally demonstrated high durability of RE-DOBDC MOFs for caustic acid gas adsorption and separation,^21,22^ it is necessary to understand the impact of fluorine incorporation in the metal cluster on gas adsorption in the framework. Therefore, metal-gas binding energies for H_2_O, NO_2_, and SO_2_ were calculated for the fully fluorinated RE-DOBDC MOF and compared to periodic DFT calculations for the hydroxyl form.

The results indicate that binding energies in the RE-DOBDC MOFs have the trend of H_2_O > SO_2_ > NO_2_, with the strongest (most negative) binding for H_2_O, consistent with previous studies in these systems.^22^ See Tables 2 and 3. Fluorination of the metal clusters results in a negligible decrease in the binding strength by 3–4 kJ/mol. Additionally, differences in the binding distances between the metal and the gas molecule are negligible (∼0.01 Å) identifying very little, if any, changes in the binding geometries. Therefore, the new revelation that fluorine exists in the metal cluster is not expected to change outcomes from previous studies.^3,22,57^

**Table 2:** Gas Binding Energies for H_2_O, NO_2_, and SO_2_ in Hydroxyl and Fluorinated RE-DOBDC MOFs

	H_2_O		NO_2_		SO_2_	
	OH	F	OH	F	OH	F
Y	–88	–83	–46	–58	–61	–57
Eu	–91	–89	–65	–67	–66	–61
Tb	–93	–89	–46	–66	–65	–62
Yb	–88	–81	–42	–54	–60	–59
avg	–90 ± 3	–86 ± 4	–50 ± 10	–61 ± 6	–63 ± 3	–60 ± 2

**Table 3:** Gas-Metal Binding Distances for H_2_O, NO_2_, and SO_2_ for Hydroxyl and Fluorinated RE-DOBDC MOFs

	H_2_O		NO_2_		SO_2_	
	OH	F	OH	F	OH	F
Y	2.46	2.46	2.52	2.56	2.57	2.58
Eu	2.49	2.49	2.55	2.55	2.62	2.62
Tb	2.46	2.46	2.50	2.60	2.57	2.59
Yb	2.41	2.41	2.46	2.53	2.55	2.56
avg	2.46 ± 0.03	2.46 ± 0.03	2.51 ± 0.04	2.56 ± 0.03	2.58 ± 0.03	2.59 ± 0.03

The fluorination of these structures could provide an advantage for gas adsorption. Examples have been synthesized that have shown advantages of selective adsorption of multiple gases for mixed-ligand MOFs,^7,60−62^ as the asymmetry of the molecule increases the pore surface area. Other possibilities for fluorination include manipulating the MOF via postsynthesis modifications,^16,61,63−65^ which would exchange the fluorine for other halogen elements. A metal cluster functional group can provide a new method for stabilizing MOF structures and another avenue for material design of MOF compositions.^33^

## 4 Conclusion

Computational studies of RE polynuclear MOFs indicate an enhanced stability in the framework through incorporation of fluorine into the metal cluster from the modulator. The existence of fluorine in the metal cluster was validated by computational and experimental ^19^F NMR spectroscopy of Y-DOBDC MOFs. ^19^F δ_iso_ peaks between −60 and −70 ppm were identified due to the unique chemical environments of fluorine. Comparison of relative peak heights between computational results at varying fluorination levels (25%, 50%, 75%, and 100%) and the experimental structure suggests a fluorination amount of ∼75% in the samples. Finally, adsorption energies for three gas molecules (SO_2_, NO_2_, H_2_O) identified limited changes in metal-gas binding energies of ∼4 kJ/mol relative to the hydroxyl structure. Overall, despite the potential for thermodynamic stability of high concentrations of fluorine in the metal cluster, the impact on the H_2_O, NO_2_, and SO_2_ gas adsorption and separation properties is minimal. Fluorine incorporation studies into other MOF metal clusters to further stabilize MOFs for acid gas separation are currently underway.

## Supporting Information Available

Computational input files, histogram of calculated ^19^F δ_iso_ chemical shift data for theY-DOBDC MOF structure, relationship between calculated Y–F bond distances and ^19^F δ_iso_ peaks in Y-DOBDC MOF structures, and the change in MOF crystal structures following fluorination (PDF)
Raw data (ZIP)
