# Supplementary material for: Dramatic Enhancement of Rare-Earth Metal–Organic Framework Stability Via Metal Cluster Fluorination
Source: JACS Au. 2022 Aug 9;2(8):1889–98. doi: 10.1021/jacsau.2c00259 (PMC9400048; doi:10.1021/jacsau.2c00259)
Supplement: Supplementary file 1 — au2c00259_si_001.pdf [file au2c00259_si_001.pdf]

Dramatic Enhancement of Rare-Earth Metal-Organic Framework Stability via Metal Cluster Fluorination

Matthew S. Christian[1], Keith J. Fritzsching[2], Jacob A. Harvey[1], Dorina F. Sava Gallis[3], Tina M. Nenoff[4,*], Jessica M. Rimsza[1,*]

1. Geochemistry Department, Sandia National Laboratories, Albuquerque, NM 87123, USA
2. Organic Materials Science Department, Sandia National Laboratories, Albuquerque, NM 87123, USA
3. Nanoscale Sciences Department, Sandia National Laboratories, Albuquerque, NM 87123, USA
4. Material, Physical, and Chemical Sciences, Sandia National Laboratories, Albuquerque, NM 87123 USA

Supporting Information

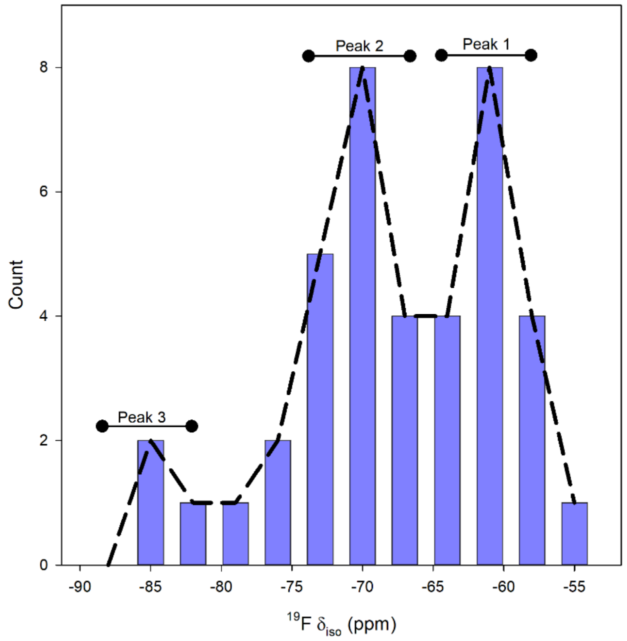

Figure S1: Histogram of calculated $^{19}F\ \delta_{iso}$ chemical shift data for Y-DOBDC MOF structure across all fluorination levels (25%-100%)

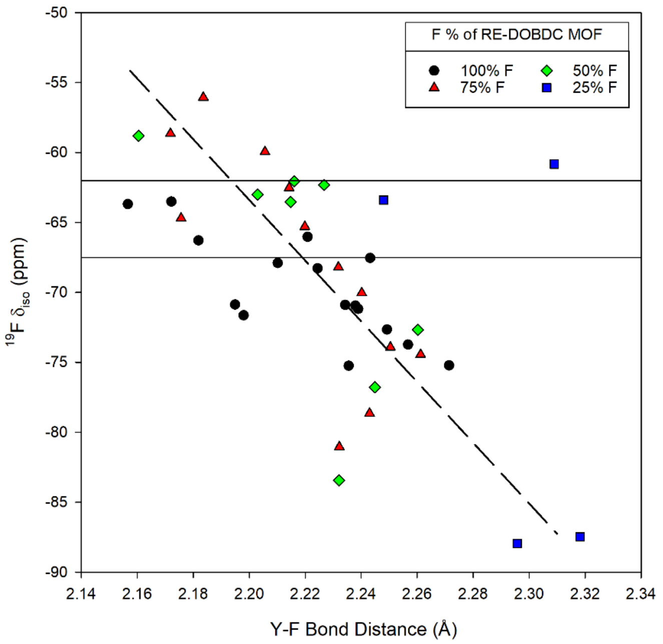

Figure S2: Relationship between calculated Y-F bond distances (Å) and $^{19}F\ \delta_{iso}$ peaks in Y-DOBDC MOF structures separated by fluorination levels. Dotted line is the linear fit of the data and the two hominization lines at -63.0 ppm and -70.0 ppm are the location of the two primary peak values.

Table S1: Volume and angles for 100% OH structures compared to experiment.

Metal	V_{DFT}	V_{EXPT}	α_{DFT}	α_{EXPT}	β_{DFT}	β_{EXPT}	γ_{DFT}	γ_{EXPT}
				UiO-66[1]				
Zr	2,230	2,232	60.000	60.000	60.000	60.000	60.002	60.000
Y	2,423	--	60.005	--	60.003	--	60.005	--
Eu	2,495	--	60.002	--	60.002	--	60.003	--
Tb	2,444	--	59.999	--	59.999	--	59.999	--
Ho	2,401	--	60.000	--	60.003	--	60.005	--
Yb	2,347	--	60.003	--	60.001	--	59.997	--
				DOBDC[2]				
Y	4,899	4,907	90.309	90.000	89.616	90.000	89.684	90.000
Eu	4,995	5,070	90.112	90.000	89.629	90.000	90.341	90.000
Tb	4,923	4,672	89.871	90.000	89.899	90.000	89.726	90.000
Ho	4,827		90.077	90.000	89.590	90.000	90.268	90.000
Yb	4,759	4,841	89.736	90.000	89.423	90.000	89.947	90.000
				TPCB				
Nd	10,611		89.311		90.138		120.227	
Eu	10,267		90.988		89.321		120.244	
Yb	10,321		90.365		89.447		119.959	

Table S2: Volume and angles for 50% F structures compared to experiment.

Metal	V_{DFT}	V_{EXPT}	α_{DFT}	α_{EXPT}	β_{DFT}	β_{EXPT}	γ_{DFT}	γ_{EXPT}
				UiO-66				
Zr	2,215	2,232	59.932	60.000	59.933	60.000	59.870	60.000
Y	2,411	--	60.071	--	60.066	--	60.136	--
Eu	2,480	--	60.031	--	60.030	--	60.118	--
Tb	2,431	--	60.027	--	60.028	--	60.133	--

Ho	2,390	--	60.048	--	60.047	--	60.129	--
Yb	2,334	--	60.062	--	60.061	--	60.135	--
DOBDC-Single								
Y	4,824	4,907	89.952	90.000	89.616	90.000	90.237	90.000
Eu	4,947	5,070	89.996	90.000	89.742	90.000	90.296	90.000
Tb	4,854	4,672	89.950	90.000	89.644	90.000	90.246	90.000
Ho	4,776		89.945	90.000	89.644	90.000	90.218	90.000
Yb	4,687	4,841	89.876	90.000	89.691	90.000	90.038	90.000
DOBDC-Double								
Y	4,738	4,907	90.033	90.000	90.174	90.000	90.094	
Eu	4,856	5,070	90.883	90.000	90.059	90.000	89.774	90.000
Tb	4,763	4,672	89.965	90.000	90.265	90.000	89.932	90.000
Ho	4,683		89.957	90.000	90.260	90.000	89.932	90.000
Yb	4,540	4,841	90.602	90.000	89.937	90.000	90.304	90.000
TPCB								
Eu	10,263		90.961		89.303		120.003	

Table S3: Volume and angles for 100% F structures compared to experiment.

Metal	V_{DFT}	V_{EXPT}	α_{DFT}	α_{EXPT}	β_{DFT}	β_{EXPT}	γ_{DFT}	γ_{EXPT}
UiO-66								
Zr	2,200	2,232	59.995	60.000	59.998	60.000	59.997	60.000
Y	2,398	--	59.989	--	59.991	--	59.992	--
Eu	2,466	--	60.008	--	60.013	--	60.005	--
Tb	2,415	--	60.005	--	60.007	--	60.006	--
Ho	2,375	--	59.992	--	60.015	--	59.992	--
Yb	2,320	--	60.014	--	59.996	--	59.995	--
DOBDC-single								
Y	4,750		90.010	90.000	89.665	90.000	90.257	90.000
Eu	4,890	5,070	89.995	90.000	89.687	90.000	90.252	90.000
Tb	4,786	4,672	89.987	90.000	89.714	90.000	90.258	90.000
Ho	4,701		90.015	90.000	89.701	90.000	90.266	90.000
Yb	4,597	4,841	89.997	90.000	89.701	90.000	90.226	90.000
TPCB								
Nd	10,451		90.726		89.389		120.211	
Eu	10,202		90.636		89.400		120.096	
Yb	10,065		90.320		89.434		120.046	

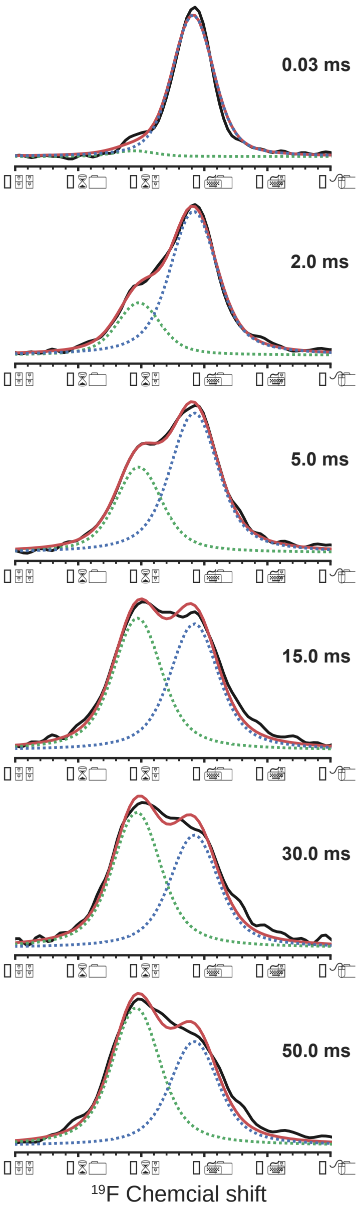

Figure S3: 2D ^{19}F-^{19}F exchange NMR data slice through F1(-69.8 ppm) (black) and fit to two Voigt peak model (red) as a function of mixing time (bold text). The dashed lines show the components of the two-peak model, the integral of the green dashed line (the cross peak) plot in Fig. 8.

References:

1. Harvey, J. A.; Greathouse, J. A.; Sava Gallis, D. F., Defect and Linker Effects on the Binding of Organophosphorous Compounds in UiO-66 and Rare-Earth MOFs. *J. Phys. Chem. C.* **2018,** *122* (47), 26889-26896.

2. Henkelis, S. E.; Vogel, D. J.; Metz, P. C.; Valdez, N. R.; Rodriguez, M. A.; Rademacher, D. X.; Purdy, S.; Percival, S. J.; Rimsza, J. M.; Page, K.; Nenoff, T. M., Kinetically Controlled Linker Binding in Rare Earth-2,5-Dihydroxyterepthalic Acid Metal-Organic Frameworks and Its Predicted Effects on Acid Gas Adsorption. *ACS Appl Mater Interfaces* **2021,** *13* (47), 56337-56347.
